# Miss Margaret B. Bowden

**Published:** 1912-09

**Authors:** 


Miss Margaret B. Bowden was found drowned in Niagara River, Aug. 5, 1912. For some time, and especially for the last year, she had been in bad health, with marked nervous prostration. She was a member of an old New England family but had resided in Buffalo for many years. At the time of her death, she was in her sixtieth year. For nearly a quarter of a century, Miss Bowden had served as secretary to the late Col. W. W. Potter, M. D., the former editor of this JOURNAL. As Dr. Potter's health declined, the active work of the JOURNAL fell more and more to Miss Bowden and for some months after his death, she was the sole proprietor, manager and editor, save for the voluntary assistance in strictly medical details, of several friends. On account of her modesty, few except these collaborators knew of the extent of her duties or realized her marked ability not only as a business woman but as a judge of strictly medical literature. Without technical instruction, she had gradually acquired a familiarity with medical journalism and was both a fluent writer and a wise critic of submitted articles. Last summer, we repeatedly urged Miss Bowden to continue in charge of the JOURNAL with such professional assistance as was volunteered but she realized her bodily and mental fatigue and refused. After this refusal, we urged that she should continue in active charge at least of the business details but were forced to yield to her firm resolution to retire completely from the work. She would not, while living, permit a tribute to be paid to her splendid executive and literary ability, her courage and devotion in carrying on the JOURNAL in the interim between editorships, and the sacrifice of health and strength which the labor involved. We feel this acknowledgement of her services to the profession of Western New York, though perhaps contrary to her own wishes, is demanded as a matter of honesty.

